# Tyrosine 870 of TLR9 is critical for receptor maturation rather than phosphorylation-dependent ligand-induced signaling

**DOI:** 10.1371/journal.pone.0200913

**Published:** 2018-07-19

**Authors:** Chhanda Biswas, Sheila Rao, Katharine Slade, David Hyman, Devin Dersh, Adriana R. Mantegazza, Philip W. Zoltick, Michael S. Marks, Yair Argon, Edward M. Behrens

**Affiliations:** 1 Division of Pediatric Rheumatology, Children's Hospital of Philadelphia, Philadelphia, Pennsylvania, United States of America; 2 Department of Pathology and Laboratory Medicine, The Children’s Hospital of Philadelphia, Philadelphia, PA, United States of America; 3 Department of Physiology, Perelman School of Medicine, University of Pennsylvania, Philadelphia, PA, United States of America; University of Tennessee Health Science Center, UNITED STATES

## Abstract

Toll like receptors (TLRs) share a conserved structure comprising the N-terminal ectodomain, a transmembrane segment and a C-terminal cytoplasmic Toll/IL-1 receptor (TIR) domain. Proper assembly of the TIR domain is crucial for signal transduction; however, the contribution of individual motifs within the TIR domain to TLR trafficking and signaling remains unclear. We targeted a highly conserved tyrosine (Y870) located in the box 1 region of the TIR domain of most TLRs, including TLR9, previously described to be a critical site of phosphorylation in TLR4. We reconstituted bone marrow-derived dendritic cells (BMDC) from *Tlr9*^*-/-*^ mice WT TLR9 or Y870F or Y870A mutants. Despite normal interactions with the luminal chaperones GRP94 and UNC93B1, Y870F conferred only partial responsiveness to CpG, and Y870A had no activity and functioned as a dominant negative inhibitor when coexpressed with endogenous TLR9. This loss of function correlated with reduction or absence, respectively, of the 80 kDa mature form of TLR9. In Y870F-expressing cells, CpG-dependent signaling correlated directly with levels of the mature form, suggesting that signaling did not require tyrosine phosphorylation but rather that the Y870F mutation conferred reduced receptor levels due to defective processing or trafficking. Microscopy revealed targeting of the mutant protein to an autophagolysosome-like structure for likely degradation. Collectively we postulate that the conserved Y870 in the TIR domain does not participate in phosphorylation-induced signaling downstream of ligand recognition, but rather is crucial for proper TIR assembly and ER egress, resulting in maturation-specific stabilization of TLR9 within endolysosomes and subsequent pro-inflammatory signaling.

## Introduction

Toll-like receptors (TLRs) are a class of pattern recognition receptors (PRRs) that recognize pathogen associated molecular patterns (PAMPs) expressed by various microbes including bacteria, viruses, fungi, protozoa, and parasites. Upon ligand recognition, TLRs signal for the production of proinflammatory and antiviral mediators and the upregulation of costimulatory molecules. These events aid in pathogen clearance by recruiting cells of the innate immune system and by stimulating pathogen-specific adaptive immunity. Mice lacking specific TLRs or adaptor molecules associated with TLR signaling have severe defects in their ability to control certain pathogens, often resulting in death, and receptor polymorphisms identified in humans have been associated with increased susceptibility to infectious and autoimmune disease [1, 2]. Therefore, investigating how TLRs function is critical to enhance our understanding of disease progression and treatment.

Prior to ligand recognition, a functional receptor must be generated and transported to the cell surface or endolysosomal compartments to initiate signaling. Like most transmembrane glycoproteins, newly synthesized TLRs such as TLR9 are generated in the endoplasmic reticulum (ER), where they undergo core glycosylation, folding, assembly into dimers, and quality control. From the ER, they must progress through the secretory pathway to their ultimate destination. TLR9 ultimately encounters its ligands–DNA enriched in unmethylated CpG from internalized viruses or bacteria–and signals from within endolysosomes. TLR9 is escorted through the secretory pathway to endosomes by its physical interaction with the membrane protein UNC93B1 [3, 4]. In the absence of UNC93B1 TLR9 fails to traffic to endosomes, and CpG-induced signaling is abolished. Within endosomes, full length TLR9 is proteolytically processed to generate its mature form [5–7]. While the unprocessed receptor is capable of binding CpG, only the processed form can interact with MyD88 to initiate downstream signaling. This prevents inappropriate signaling by limiting the signaling-competent receptor to only endolysosomal compartments. Additionally, in some cell types, trafficking of this receptor to endosomal compartments or phagosomes ensures that TLR9 is in proximity with the appropriate signaling molecules [8, 9].

**Fig 1:**
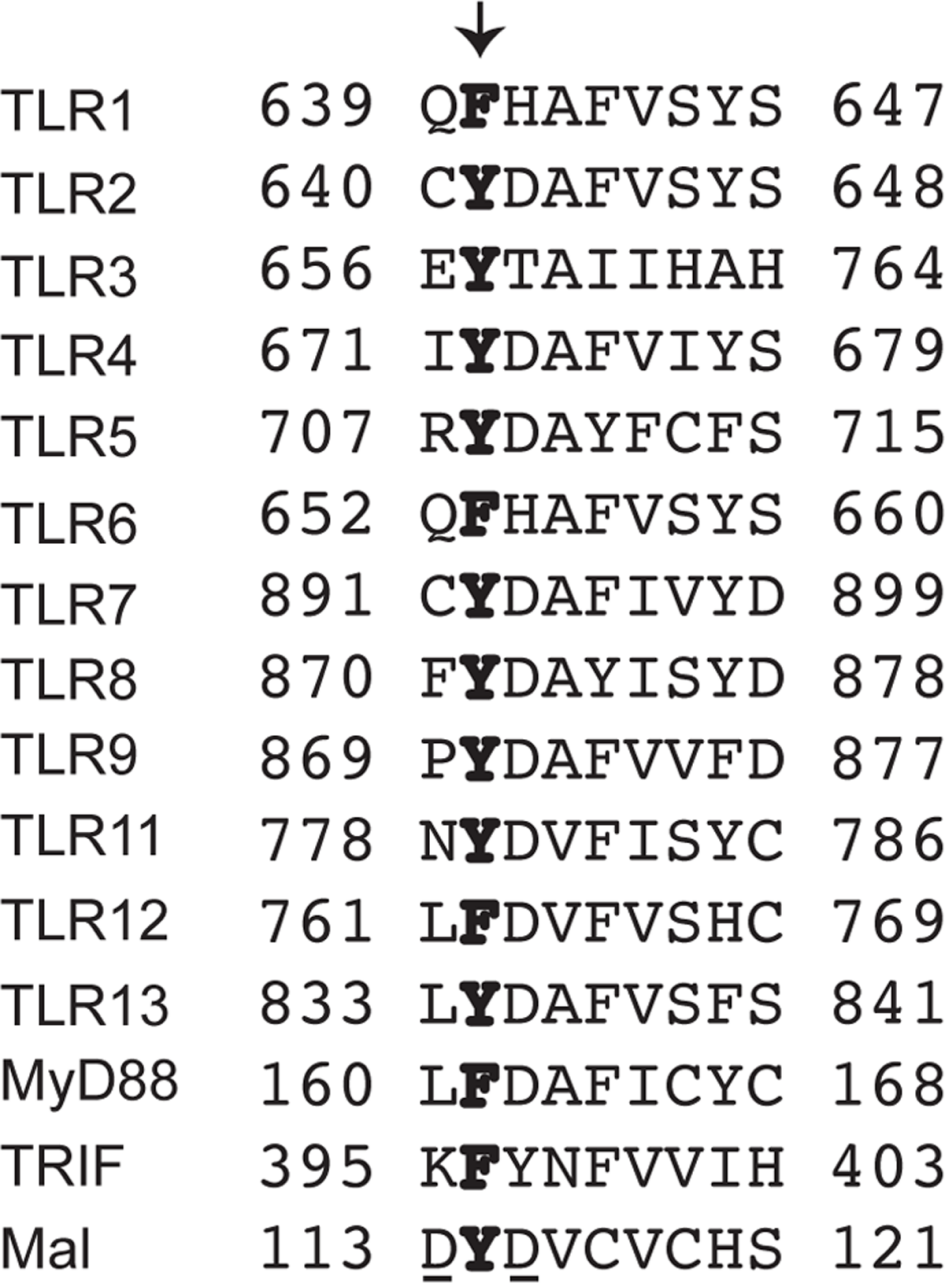
TLR9 tyrosine 870 is conserved across TLRs and their adaptors. Mouse TLRs, except TLR1, TLR6, and TLR12, have a tyrosine in the box 1 region of the TIR domain. N-terminal and C-terminal amino acid positions are noted to the left and to the right respectively. Note the aspartic acid residues in Mal that are underlined were found to be potentially involved in the dimerization surface in the crystal structure [15].

All TLRs contain a conserved cytoplasmic Toll/interleukin-1 receptor (TIR) domain that facilitates interactions with other TLRs and the TIR domain-containing adaptor molecules TRIF and MyD88 [10–12]. The TIR domain is comprised of about 160 amino acids and is essential for downstream signaling. The domain contains three short highly conserved regions, termed box 1 (F/Y)DA, box 2 RDXXPG, and box 3 FW, all of which were reported to be important for receptor function based on analyses of a series of alanine substitution mutants [13]. Interestingly, the tyrosine residue of box 1 is conserved among all TLRs except TLRs 1, 6, and 12 (Fig 1). These receptors instead contain a phenylalanine in this position. Moreover, these are the only TLRs that are not observed as homodimers. TLRs 1 and 6 form heterodimers with TLR2, and TLR12 forms heterodimers with TLR11. These observations suggest either that tyrosine phosphorylation of the box 1 tyrosine residue is necessary for full receptor activation, as has been suggested for TLR4 [14], or that this residue is critical for the structural integrity of the TIR domain during dimerization. To distinguish between these possibilities, we generated TLR9 mutants containing either phenylalanine or alanine at residue 870. We find that this residue likely does not participate in phosphorylation-induced signaling downstream of ligand recognition. Our data demonstrate that the conserved tyrosine residue in the box 1 region of the TIR domain is critical for proper TLR9 assembly and targeting to endosomes, and that mutagenesis of this residue causes degradation of TLR9 by autophagy. These data clarify published studies in which it was suggested that phosphorylation of this residue is critical for TIR domain signaling of TLR4. We propose that this conserved tyrosine dictates receptor stability in the ER for other TLRs rather than participating in ligand-induced signaling.

## Results

### Mutagenesis of TLR9 tyrosine 870 results in defective CpG-induced signaling

**Fig 2:**
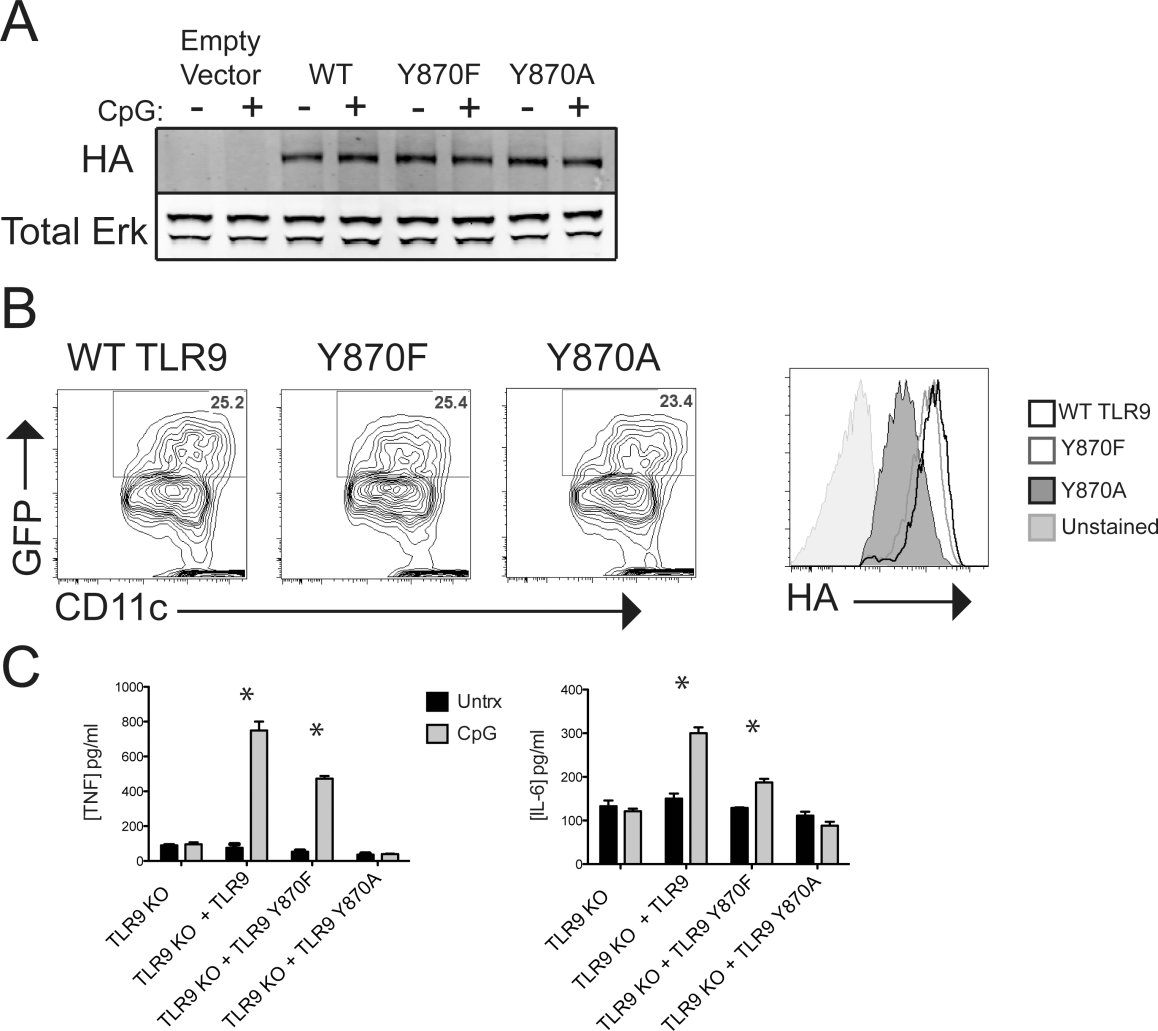
Expression of HA-tagged TLR9 mutants in 293 T cells and BMDCs. (A) 293T cells were transfected with 4μg of HA-tagged WT TLR9, Y870F, or Y870A. Cells were untreated (-) or treated for 18 h with 1 μg/ml CpG, and whole cell lysates were fractionated by SDS-PAGE and analyzed by Western blot for HA (TLR9) or Erk as a loading control. Blots were developed using Odyssey and analyzed by Licor software. The data shown are representative of at least 4 independent experiments. (B) *Tlr9*^*-/-*^ bone marrow was transduced with retrovirus expressing HA-tagged WT TLR9, Y870F, or Y870A. BMDCs were harvested, permeabilized and stained with antibodies against CD11c and HA. HA expression (right panel) was determined for GFP+CD11c+ cells (left panels) by flow cytometry. Histograms are representative of at least 5 independent experiments. (C) *Tlr9*^*-/-*^ BMDCs expressing WT TLR9, Y870F, or Y870A were left unstimulated (Untrx) or stimulated for 3 hours with CpG (1μg/ml). Supernatants were harvested, and TNFα and IL-6 were measured by ELISA. Bar graphs are representative of at least 4 independent experiments performed in triplicate. * indicates p-value < 0.05 by Bonferroni post-test.

To investigate the effects of tyrosine 870 on CpG-induced TLR9 function and signaling, we generated recombinant retroviruses expressing TLR9 with either the native tyrosine at position 870 (wild-type or WT), a phenylalanine (Y870F) as in TLR1, 6 and 12, or an alanine (Y870A). All TLR9 variants had a cytosolic HA tag for detection. The retroviral transcripts also drive expression of green fluorescent protein (GFP) from an internal ribosome entry site. Immunoblotting analysis of 293T cells transduced with viruses encoding WT TLR9, Y870F, and Y870A showed that each was expressed as a full-length protein (Fig 2A). Upon transduction of each recombinant retrovirus into bone marrow from *Tlr9*^*-/-*^ mice and subsequent differentiation into bone marrow-derived dendritic cells (BMDCs), GFP-positive cells expressed intracellular HA, although Y870A was expressed at lower levels than WT or Y870F (Fig 2B). CpG stimulation of *Tlr9*^*-/-*^ BMDCs expressing WT TLR9 resulted in robust secretion of TNFα and IL-6 as expected (Fig 2C). By comparison, TNFα and IL-6 secretion was reduced from cells expressing the Y870F mutant, and cells expressing Y870A did not secrete cytokine above background in response to CpG (Fig 2C). These data suggest that TLR9 tyrosine 870 is important for CpG-induced signaling.

### Expression of TLR9 Y870A inhibits ligand induced cytokine secretion by endogenous TLR9 in a dominant-negative manner

**Fig 3:**
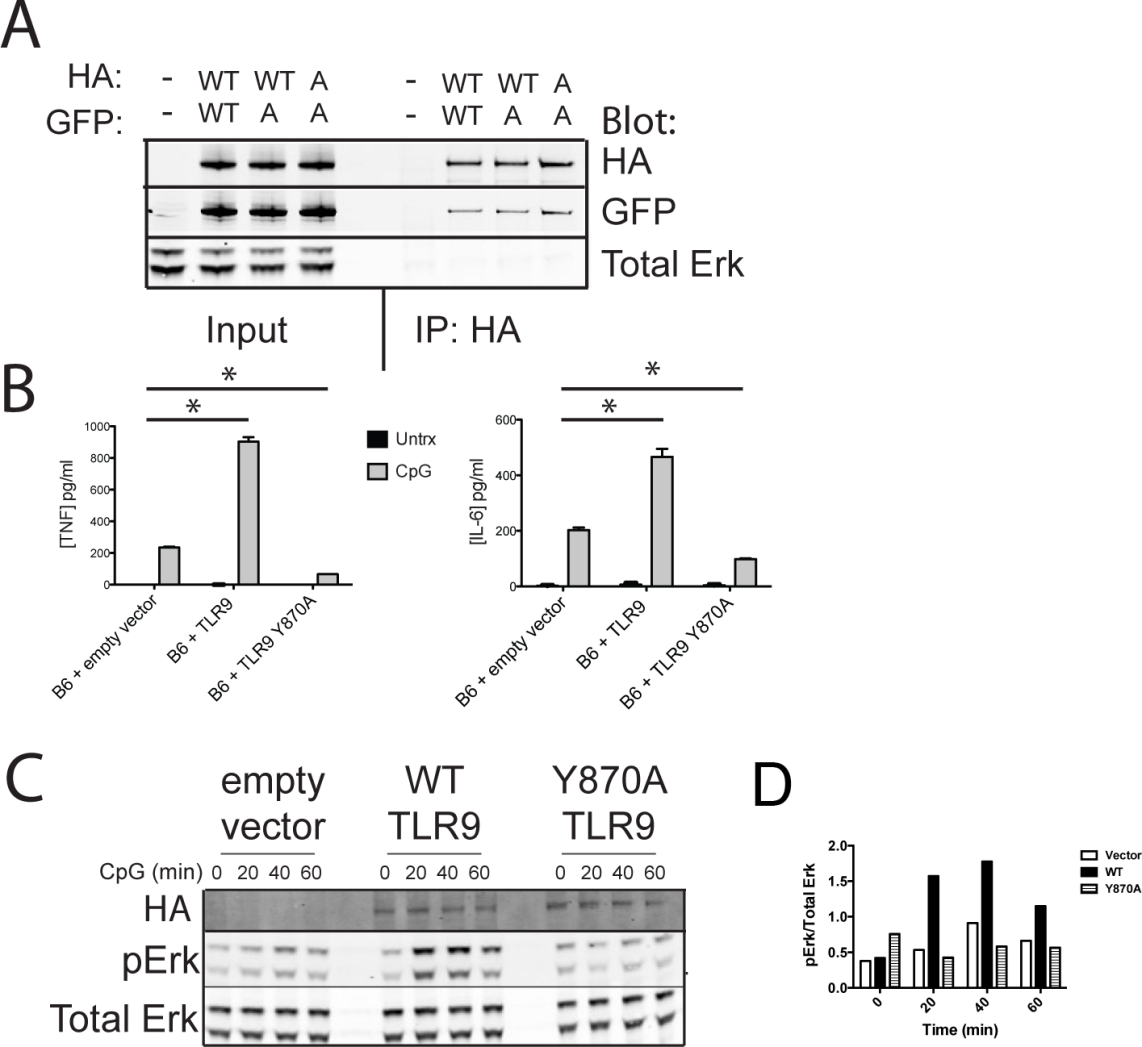
Expression of TLR9 Y870A inhibits ligand induced cytokine secretion by endogenous TLR9 in a dominant-negative manner. (A) 293T cells were co-transfected with HA- and GFP-tagged forms of TLR9 as WT (WT) or Y870A (A) variants as indicated. HA-tagged TLR9 was immunoprecipitated using an antibody to HA, and interaction with GFP-tagged TLR9 was assessed by immunobloting for both HA and GFP in both the lysates (input) and the immunoprecipitates (IP: HA). Immunoblotting for Erk served as a control. Results are representative of at least 3 independent experiments. (B) WT (i.e., TLR9 sufficient) C57BL/6 bone marrow was transduced with retrovirus expressing HA-tagged WT or Y870A TLR9 or empty vector as a control, and then differentiated to DCs. BMDCs were stimulated for 3 hours with CpG, and then supernatants were harvested and TNFα and IL-6 were measured by ELISA. Bar graphs are representative of at least 3 independent experiments each performed in triplicates. * indicates p-value < 0.05 by Bonferroni post-test. (C) These same transduced BMDCs were stimulated with CpG for various times, and lysates were subjected to Western blot analysis for the presence of HA-tagged TLR9, phosho-Erk or total Erk. Blots are representative of 3 experiments. (D) Quantification from a representative experiment of phospho-Erk normalized to total Erk.

Because TLR9 forms homodimers [16] resulting in two TIR domain chains each containing a tyrosine at position 870, we considered the possibility that the Y870A variant is unable to dimerize. To test this, we co-expressed HA- and GFP-tagged forms of WT TLR9, the Y870A variant, or WT and Y870A together in 293T cells and probed for coimmunoprecipitation of the GFP-tagged variant in anti-HA immunoprecipitates. These experiments revealed that GFP-tagged Y870A interacted with HA-tagged WT or Y870A TLR9 (Fig 3A), indicating that Y870A does not impair dimerization. Interestingly, expression of TLR9 Y870A in C57BL/6 BMDCs, which express endogenous TLR9, led to a reduction of TNFα and IL-6 secretion in response to CpG compared to BMDCs transduced with empty vector (Fig 3B). Furthermore, CpG-induced Erk phosphorylation, which was observed 20, 40, and 60 min after stimulation in WT TLR9 transduced cells, was actually reduced in C57BL/6 BMDCs expressing Y870A compared to those transduced with empty vector (Fig 3C and 3D). These results indicate that Y870A functions as a dominant negative inhibitor and suggest the possibility that Y870A interacts with WT TLR9 and inactivates it.

### Mutagenesis of tyrosine 870 results in defective receptor maturation

**Fig 4:**
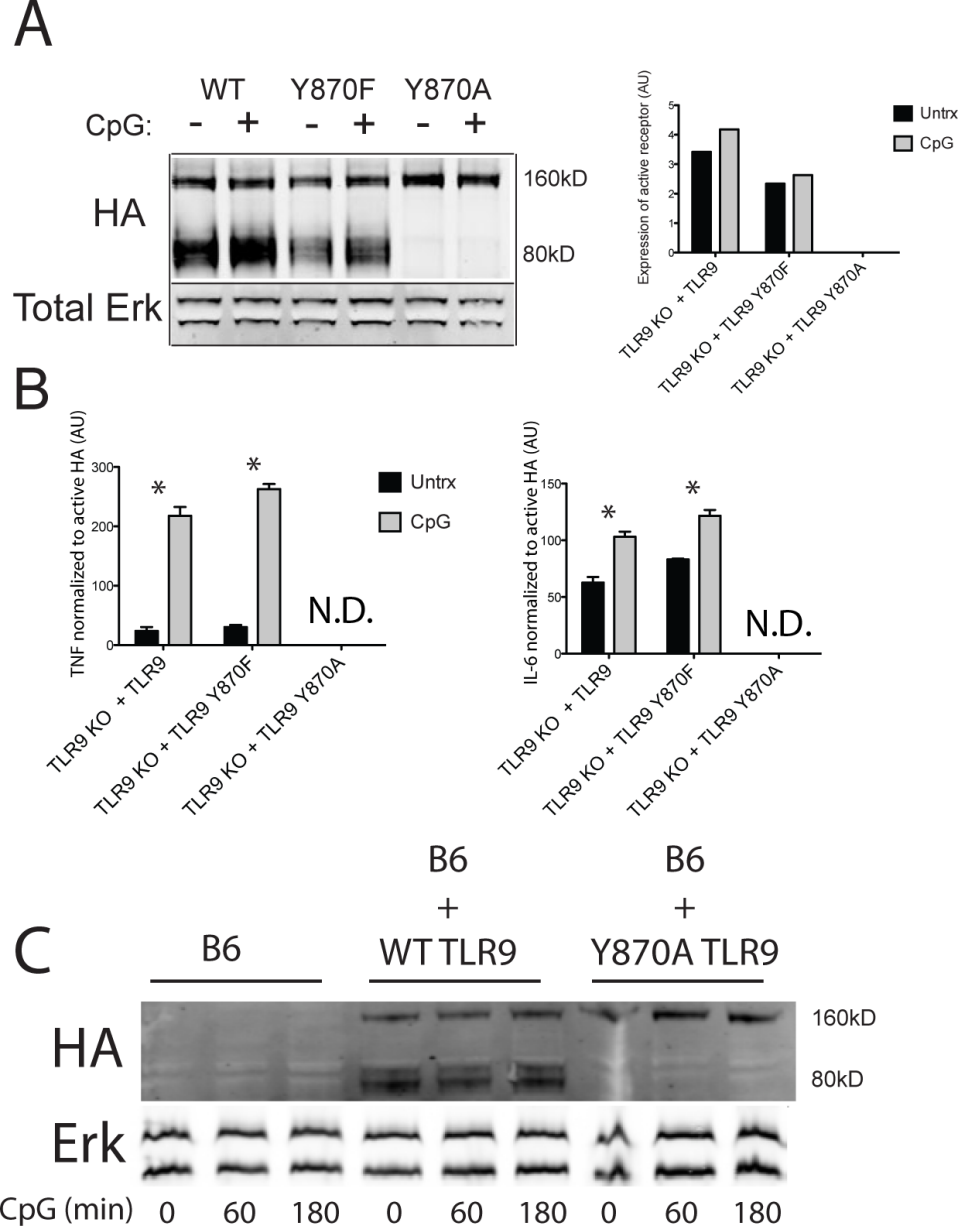
Mutagenesis of TLR9 Tyr870 results in impaired receptor maturation. (A, B) *Tlr9*^*-/-*^ BMDCs expressing HA-tagged TLR9 WT, Y870F, or Y870A were untreated (-) or treated for 18 h with 1 mg/ml CpG. (A) Cells were lysed and analyzed by Western blotting for HA expression relative to Erk as a loading control. A representative blot from 3 experiments is shown at the left. Right, the 160 kDa and 80 kDa bands were quantified, and the amount of active receptor was calculated by normalizing the amount of processed receptor (80 kDa) to the amount of unprocessed receptor (160 kDa). Shown is the average from 3 experiments. (B) CpG-induced TNFα and IL-6 levels from WT or mutant TLR9-expressing BMDC cultures were normalized to the amounts of processed (active) receptor as determined in Fig 4A. N.D. indicates not determined, as there is no mature Y870A receptor to which cytokine can be normalized. Note, CpG-induced cytokine levels in Y870A-expressing cells were essentially no different from unstimulated samples, as in Fig 2C. * indicates p-value < 0.05 by Bonferroni post-test. (C) WT bone marrow was uninfected (left) or transduced with either WT or Y870A TLR9, and then differentiated to BMDCs. Cells were stimulated for 0, 60 or 180 min with CpG as in Fig 3B, and cleavage of overexpressed TLR9 to the 80kD form in these cells was assessed by immunoblotting for HA (relative to total Erk as a loading control) as in (A). Shown is a representative of 3 experiments.

TLR9 is proteolytically processed within endosomes to generate its mature form [5, 7]. Controversy exists as to whether the cleaved N-terminal portion is required to form a tri-molecular complex with ligand and the C-terminal portion for signaling [17, 18], calling into question whether the cleaved C-terminal TLR9 molecule alone is sufficient for TLR9 ligand dependent function. Furthermore, in some scenarios including ectopic TLR9 expression [17, 18] and mutated transmembrane domain TLR9 [19], full length TLR9 seems capable of signaling, suggesting cleavage is not even necessary. However, it is typically accepted that endogenously expressed TLR9 needs to undergo a cleavage event, only after post-translational glycosylation and trafficking to the endosome, in order to signal after ligand binding [17, 18]. Because Y870A does not signal, we considered the possibility that mutation prevents proteolytic cleavage. Indeed, whereas the mature, processed form of TLR9 (80kD) was readily observed in *Tlr9*^*-/-*^ BMDCs that were transduced with WT TLR9, cleavage was not observed in cells expressing the Y870A variant, even after stimulation with CpG (Fig 4A). Furthermore, cleavage of TLR9 Y870A was not rescued when expressed in BMDCs generated from C57BL/6 mice expressing endogenous TLR9 (Fig 4C). Together with the observation that WT and Y870A heterodimerize (Fig 3A), the data suggest that a single TLR9 chain containing a tyrosine at position 870 within a TLR9 dimer is insufficient for proper processing.

Full length Y870F and WT TLR9 were expressed at similar levels, but the expression level of mature Y870F was decreased compared to WT TLR9 (Fig 4A). In fact, by normalizing the quantity of CpG-induced secreted cytokine (Fig 2) to the expression level of the processed form of the receptor, stimulation of mature WT and Y870F TLR9 elicited comparable levels of TNFα and IL-6 release (Fig 4B). Collectively, these data indicate that TLR9 tyrosine 870 is necessary to maximally generate a fully processed, mature receptor, and that a phenylalanine in this position impairs processing but is sufficient for signaling. Because essentially no mature receptor is generated when this residue is an alanine, it is difficult to directly assess the signaling capabilities of Y870A in this system.

### Y870 is necessary for receptor stabilization

**Fig 5:**
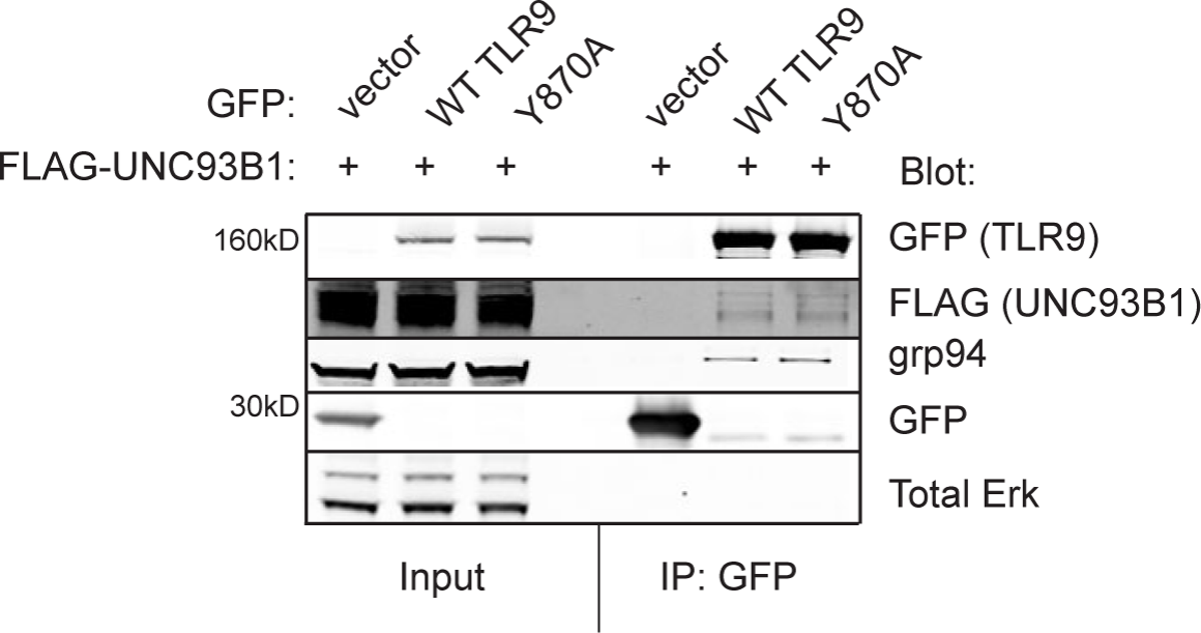
TLR9 Y870A interacts with ER resident proteins. 293T cells were transfected with 4μg each of FLAG-tagged UNC93B1 and GFP-tagged WT or Y870A TLR9 or a GFP vector control. Lysates were harvested and subjected to immunoprecipitation with anti-GFP-conjugated agarose beads. Aliquots of the lysate (input) and immunoprecipitates (IP: GFP) were analyzed by Western blotting for GFP (to detect ~160kDa TLR9 transgene or ~30 kDa GFP control), FLAG (to detect UNC93B1 transgene), GRP94 (to detect associated endogenous GRP94) or Erk as a control. Data are representative of 3 independent experiments.

We sought to explore if the impaired maturation of Y870A is due to a defect in endolysosomal proteolytic processing or inappropriate trafficking of the receptor such that it does not reach endosomal compartments. We first tested whether the mutant TLR9 is able to interact with the appropriate chaperones during folding in the ER. GRP94 is a resident "master" chaperone of TLRs, including TLR9, and GRP94-deficient cells do not respond to CpG [20–23]. When expressed in 293T cells, both GFP-tagged WT TLR9 and Y870A interacted with endogenous GRP94 as assessed by coimmunoprecipitation (Fig 5), suggesting that the Y870A mutation does not impair normal lumenal chaperone interactions. Both WT and Y870A TLR9 interact with the ER chaperone protein UNC93B1 (Fig 5), which escorts TLR9 from the ER to endosomes and is necessary for CpG-induced signaling [3, 4]. Thus the Y870A variant can interact with the appropriate chaperone molecules in the ER and does not likely impact lumenal ER folding.

**Fig 6:**
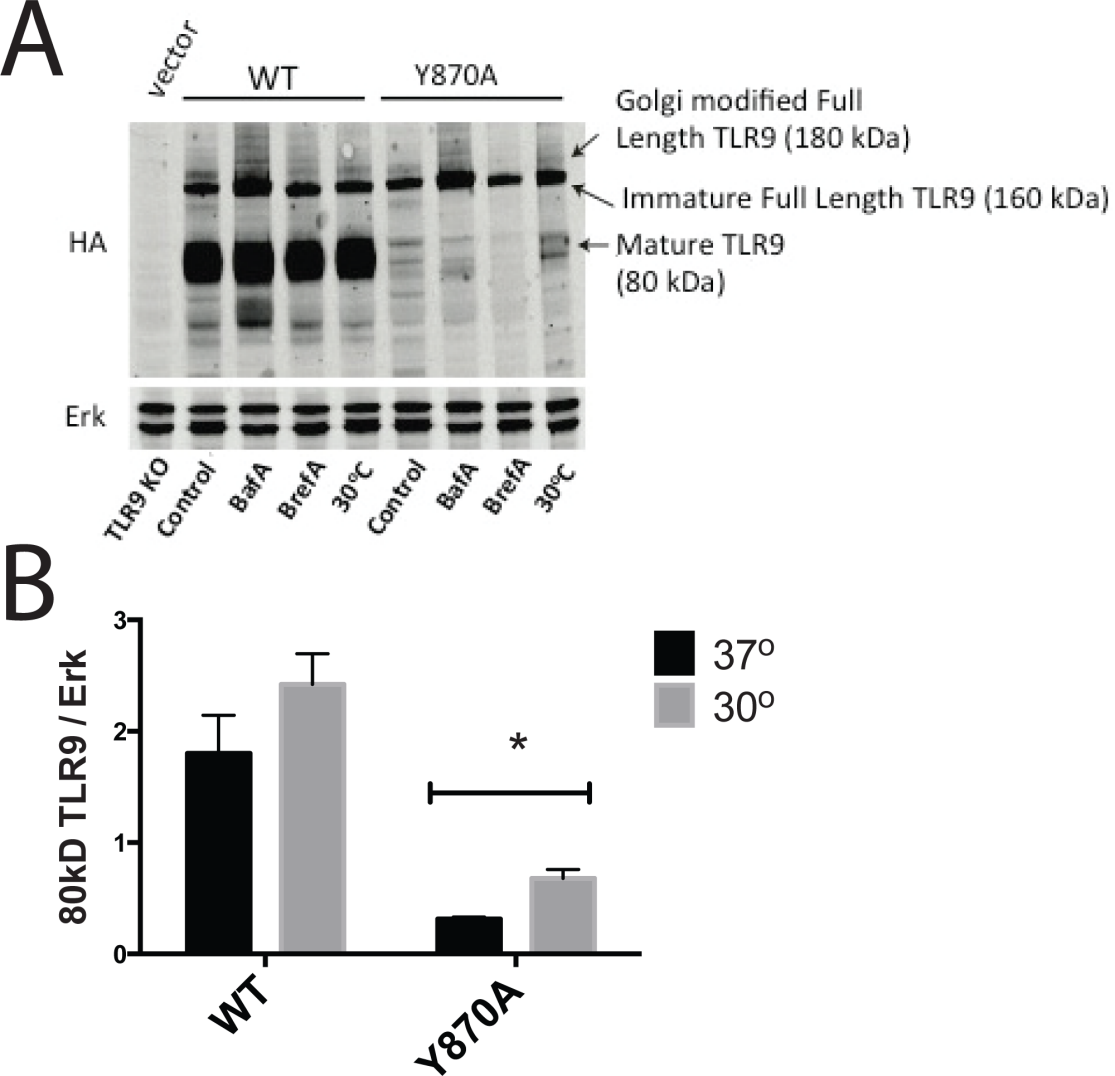
Defective egress of Y870A TLR9 from the ER results in an inability to generate active receptor. (A) *Tlr9*^*-/-*^ bone marrow was transduced with WT or Y870A TLR9 and differentiated to DCs. BMDCs were cultured at 37°C in the absence or presence of Baf A (0.5 μM) or Bref A (7 nM) for 4 hrs, or at 30°C for 18 hrs. Cell lysates were then assessed for TLR9 processing by immunoblotting for HA. Erk is shown as a loading control, and untransduced *Tlr9*^*-/-*^ BMDCs (TLR9 KO) are shown as a negative control. Blot shown is representative of 3 independent experiments. Positions of bands representing the full length TLR9 precursor (160 kDa), the Golgi-modified full-length TLR9 (~180 kDa), or mature processed TLR9 (80 kDa) are indicated. B. Quantification of the mature 80 kDa TLR9 product normalized to Erk at 37 and 30 degrees conditions averaged from 3 experiments. * p < 0.001.

The finding that the Y870A variant interacts with lumenal ER resident proteins does not preclude a defect in ER exit due to misfolding of the cytoplasmic domain. Nor does it preclude the possibility of Y870A exiting the ER; it is possible that Y870A is not targeted appropriately to endosomes or is improperly cleaved within endosomes and subject to lysosomal degradation, thus potentially explaining the reduced levels of mature Y870A relative to WT TLR9. To test the latter possibility, we treated cells expressing WT or Y870A TLR9 with the proton ATPase inhibitor, BafilomycinA (Baf A), which blocks lysosomal acidification and thereby impairs lysosomal protein degradation. Compared to untreated cells, cells treated for 4 hours with Baf A accumulated the full length, glycosylated forms of both WT and Y870A TLR9 (Fig 6). However, the degree to which full length Y870A accumulated was not greater than that of full length WT TLR9. Moreover, Baf A treatment did not "rescue" the mature cleaved form of Y870A (Fig 6). These data suggest that Y870A is not targeted to lysosomes and subsequently degraded to a substantial degree, and that proteolytic maturation is not masked by degradation.

We therefore next considered the possibility that Y870A is not processed because it does not fold properly to a stable protein. We observed a reduced-intensity band that migrates slightly more slowly than the predominant full-length TLR9 (Fig 6), suggesting reduction in normal post-ER trafficking and subsequent Golgi processing of Y870A relative to WT TLR9 expressed in transduced *Tlr9*^*-/-*^ BMDCs. This band represents the Golgi-modified form of full-length TLR9 with mature N-linked oligosaccharides [6], since it was eliminated from WT TLR9-expressing cells that were treated for 4 hours with brefeldin A (Bref A), which inhibits trafficking to the Golgi (Fig 6). We then tested whether normal post-ER trafficking, and thus proteolytic processing, were improved by growth at reduced temperature, which often helps to stabilize partially folded proteins. Indeed, whereas levels of full-length WT-TLR9 in transduced *Tlr9*^*-/-*^ BMDCs were not affected by growth at 30°C, levels of full-length Y870A and its Golgi-modified form were slightly increased by growth for 18 hours at 30°C (Fig 6). A modest accumulation of the mature 80 kDa form was also observed, suggesting that cold induced stabilization improved targeting of the mutant protein to endosomes.

**Fig 7:**
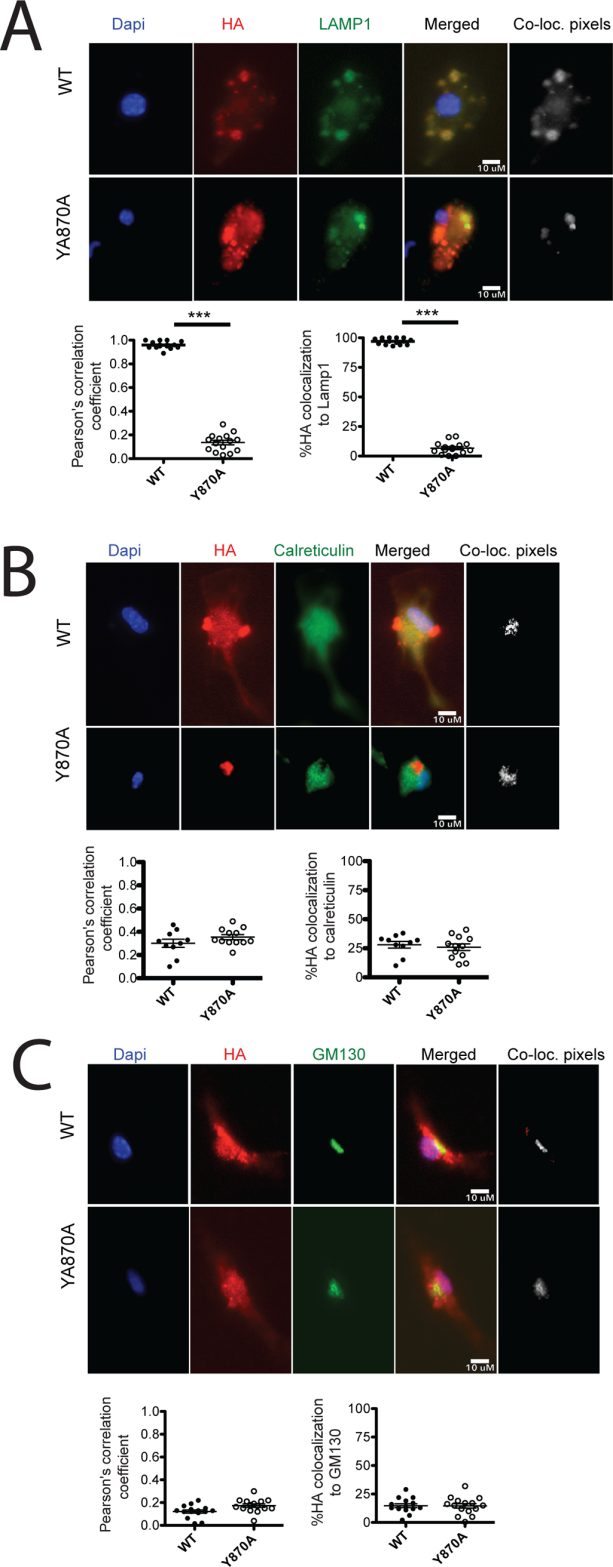
Y870A TLR9 is not retained in the ER or Golgi. *Tlr9*^*-/-*^ bone marrow was transduced with doxycycline inducible WT or Y870A HA-tagged TLR9, and differentiated towards DCs. Three days later, protein expression was induced by doxycycline (0.5μg/ml) treatment, and BMDCs were fixed and permeabilized at 4 hours post-Doxycycline treatment. The 4 h post-doxycycline fixed cells were labeled for HA and either the late endosome/lysosome marker LAMP1 (A), the ER marker Calreticulin (B), or the Golgi marker GM130 (C). In all cases, nuclei were labeled by DAPI. Each panel shows an image of both WT and Y870A-expressing BMDCs for each individual label, a merged image (Merged), and a colocalization image in which areas of overlap of the two markers, after thresholding by the method of Costes [24], is indicated by the white areas (“co-loc. pixels”). HA colocalization with each organelle marker is expressed both as a Pearson’s correlation coefficient and by the percent of manually thresholded HA-labeled structures that overlap with the marker. *, p<0.01; **, p<0.005; ***, p<0.0001.

**Fig 8:**
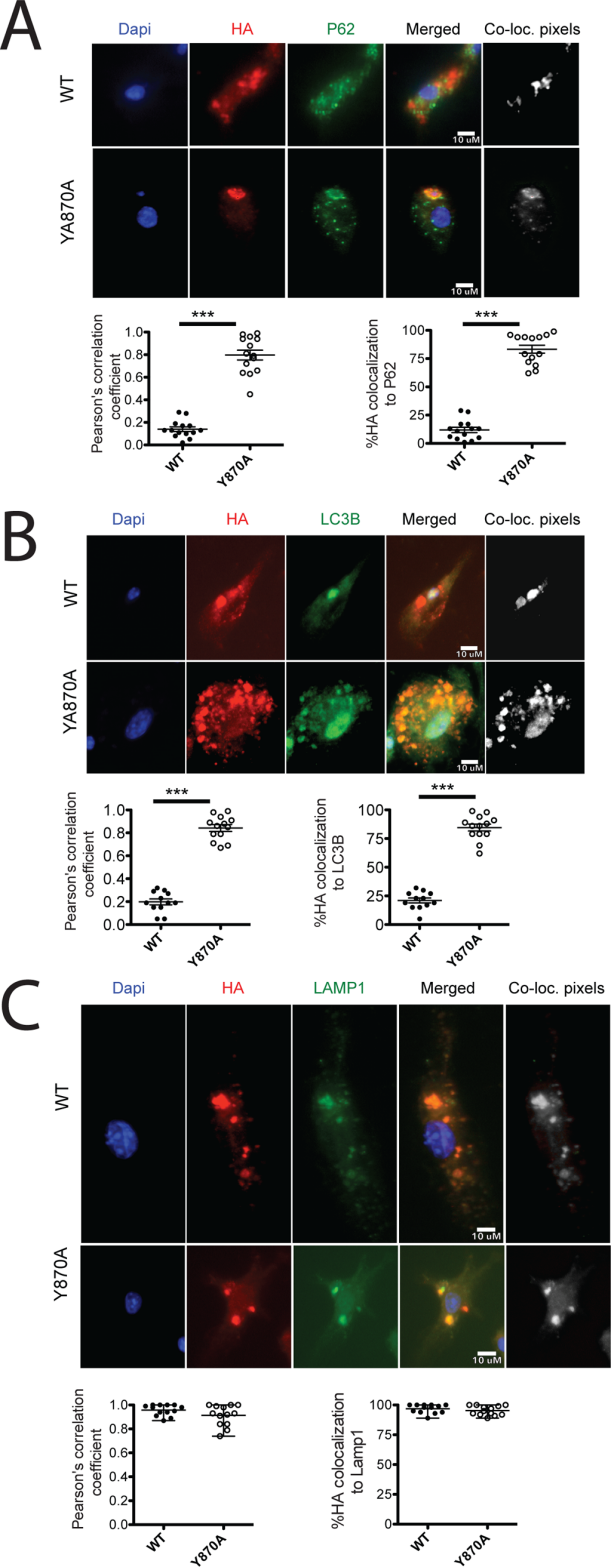
Y870A TLR is consumed by autophagy. *Tlr9*^*-/-*^ bone marrow was transduced with doxycycline inducible WT or Y870A HA-tagged TLR9, and differentiated towards DCs. Three days later, protein expression was induced by doxycycline (0.5μg/ml) treatment, and BMDCs were fixed and permeabilized at 4- and 24- hours post-doxycycline treatment. The 4 h post-doxycycline fixed cells were labeled for HA and the autophagosome markers SQSTM1 / p62 (A) or LC3B (B), and then by species-specific Alexa568- and Alexa488-conjugated secondary antibodies. The 24 h post-doxycycline fixed cells were labeled for HA and LAMP1 (C). In all cases, nuclei were labeled by DAPI. Each panel shows an image of both WT and Y870A-expressing BMDCs for each individual label, a merged image (Merged), and a colocalization image in which areas of overlap of the two markers after thresholding by the method of Costes is indicated by the white areas (“co-loc. pixels”). HA colocalization with each organelle marker is expressed both as a Pearson’s correlation coefficient and by the percent of manually thresholded HA-labeled structures that overlap with the marker. *, p<0.01; **, p<0.005; ***, p<0.0001.

Finally, we assessed by microscopy whether Y870A protein was directed to a degradative compartment rather than to signaling endolysosomes, resulting in degradation of the receptor. To assess protein localization without concern for overexpression artifacts, we generated doxycycline inducible vectors for tunable expression of HA-tagged WT and Y870A TLR9 transduced into *Tlr9*^*-/-*^ BMDCs. We fixed cells 4 hours after doxcycline induction to examine the location of newly synthesized protein expressed at moderate levels. As expected, most of the WT HA-TLR9 localized to late endosomes/ lysosomes, indicated by nearly complete colocalization with LAMP1. By contrast, although Y870A HA-TLR9 largely distributed to punctate structures throughout the cytoplasm, very few of these puncta colocalized with LAMP1 (Fig 7A). This indicates that the Y870A mutant does not localize properly to endolysosomes. Surprisingly, the puncta labeled by HA-TLR9 Y870A did not overlap with either the ER marker calreticulin (Fig 7B) or the Golgi marker GM130 (Fig 7C). These data suggest that the Y870A mutant protein is not retained in these locations, but rather accumulates in another intracellular compartment. Interestingly, puncta labeled by Y870A HA-TLR9, but not WT TLR9, overlapped strikingly with autophagosome components LC3B (Fig 8A) and p62 (Fig 8B). These data suggest that Y870A is consumed by autophagy. Consistently, 24 hours after doxycycline induction, most of the Y870A mutant accumulated in a single large perinuclear compartment that was labeled by LAMP1 (Fig 8C), indicative of autophagosome fusion with lysosomes to generate an autolysosome. By contrast, WT TLR9 remained in peripheral puncta that were also labeled by LAMP1. Taken together, these results suggest that the Y870A mutant is consumed by autophagy and subsequent degradation within autolysosomes, and is never properly targeted to endolysosomes for maturation to the 80 kDa form.

## Discussion

TLRs contain a conserved TIR domain with regions of varying homology. The tyrosine located in the box 1 region of the TIR domain is conserved among most TLRs (Fig 1). Curiously, the receptors that heterodimerize to function–TLR1, TLR6, and TLR12 –contain a phenylalanine instead of a tyrosine, but the TLRs with which these molecules pair all have a tyrosine at that position. The remaining TLRs that harbor a tyrosine form homodimers. Thus, all TLR dimers have at least one box 1 region containing the conserved tyrosine, suggesting the importance of this residue for TLR function. It was previously reported that the box 1 tyrosine was critical for TLR4 signaling [14], but how it regulated signaling or whether it also functioned in other TLRs was not determined. To more comprehensively understand the molecular components in TLR9 function, we questioned whether Y870 was important for full receptor activation in TLR9. Here, we demonstrate that substitution of Y870 for alanine results in a TLR9 variant whose maturation and subsequent signaling are abolished. Consistent with a prior report in bone marrow derived macrophages [25], we find that tyrosine at position 870 is necessary for full TLR9 stabilization and normal trafficking to endosomal compartments. We expand upon these ideas by showing that Y870 mutants can interact with the co-expressed wild type protein and that mutants harboring an alanine at this position are consumed by autophagy. It is likely that dimerization with the mutant and partially misfolded TLR9 results in some degradation of wild type molecules, therefore likely explaining the dominant negative effect of the mutant. While activated TLR9 has been shown to be targeted for autophagy upon B-cell receptor signaling in B cells [26], we now demonstrate that a mutant TLR9 can be similarly targeted in myeloid cells independent of any signaling.

Mutagenesis of the analogous N-terminal box 1 tyrosine residue in TLR4 (Y674A) abolished LPS-induced signaling in a heterologous expression system in 293T cells [14]. While the investigators attributed the defective signaling to decreased LPS-induced receptor phosphorylation, our data suggest the alternative possibility that the receptor did not traffic normally to the plasma membrane to access ligand. In fact, our observation that TLR9 Y870F signals as effectively as WT TLR9 in response to CpG in dendritic cells, after correcting for differences in levels of mature TLR9 (Fig 4B), corroborates previously published data in bone marrow derived macrophages [25] and indicates that a tyrosine at the N-terminal box 1 position is not strictly necessary for CpG-induced signaling, but rather supports generating a functional receptor. Furthermore, these observations indicate that receptor phosphorylation at this site is not necessary for function, as was suggested for TLR4 [14]. Importantly, the TLR4 report did not investigate signaling with a phenylalanine mutant. Therefore, it is possible that in their report, as we see here for TLR9, TLR4 was retained in a compartment without access to ligand, explaining the apparent loss of activity. Our data support this notion for TLR9 and suggest that the conserved residue is necessary for receptor stabilization. Alternatively, this residue may be important for interaction with cytoplasmic chaperone molecules important for TLR folding and/or localization. Since Y870 is within the cytoplasmic TIR domain of TLR9, our data are consistent with a requirement for heretofore undiscovered cytoplasmic chaperones in TLR folding, analogous to the requirement for both ER-resident and cytoplasmic chaperones for folding of the cystic fibrosis transporter CFTR [27]. Hsp70 family members play an important role in cytoplasmic folding for CFTR and could be playing a similar unappreciated role in TLR folding as well.

The cytoplasmic TIR domain of all TLRs is best known for its ability to interact with the adaptor molecules TRIF/TRAM and/or MYD88/TIRAP to transduce downstream signaling following ligand engagement [28]. An additional role for localization was attributed to this region, as a TLR9 molecule with deletions of several amino acids in the TIR domain downstream of Y870 was mislocalized to the cell surface [29]. In contrast, our data suggest that point mutation of the N-terminal tyrosine 870 in the Box 1 of the TIR domain prevents normal TLR9 egress from the ER. One possible explanation for this discrepancy could be attributed to the cell types used for experimentation. We conducted our studies in BMDCs, which more closely resemble physiologically relevant TLR-responsive innate immune cell types than do HeLa cells that were used in other studies [29]. It is possible that non-physiologic, cell line-specific machinery contributed to the trafficking of overexpressed TLR9 in the HeLa experiments. Nonetheless, both pieces of evidence support the concept that TIR domain assembly is important for the correct sub-cellular localization and, thus, function of TLR9.

Previous work has suggested that some residues of the box 1 region of the TIR domain create a binding pocket required for TIR-TIR domain interactions between the TLR and its adaptor Mal/TIRAP [15]. Our data are consistent with a model in which the N-terminal tyrosine of this domain is critical for proper TIR domain assembly and ER egress. Accordingly, the more conservative Y870F mutation has an intermediate phenotype, while the more divergent Y870A mutant completely disrupts proper assembly. Interestingly, the Y870A variant forms homodimers, the interaction for which is based on the N-terminal ectodomain [16], and associates with its appropriate ER chaperone molecules GRP94 and UNC93B1. These data suggest that altered ER egress does not reflect misfolding in the lumenal space. Rather, these data are consistent with a model in which misfolding of the TIR domain is recognized by a cytoplasmic quality control system [30] and is sufficient to prevent normal ER egress. A properly folded TIR domain is likely partially stabilized by growth at 30°C, permitting some degree of normal ER exit—even for the mutant forms—to eventually reach endosomes. Therefore, we propose a model of TLR processing that requires proper TIR domain assembly prior to ER exit and subsequent subcellular localization. Interestingly, the observed dimerization of mutant TLR9 must take place in the ER since the mutant TLR9 is unable to exit the ER to endosomes. This suggests that TLR9 dimerization via the luminal domain is a ligand independent process, as these molecules would not be expected to have access to their ligands within the ER.

It is of interest that the Y870A TLR9 molecule is consumed by autophagy for disposal, rather than exploiting the endoplasmic-reticulum-associated protein degradation (ERAD) pathway. ERAD makes use of ubiquitinylation and proteosomal degradation rather than autophagy. It has been proposed that proteins that form large aggregates, too large for the pore of the proteasome machinery, are instead degraded by autophagy [31, 32]. Disposal of improperly folded transmembrane proteins by autophagy has been described [31] but is relatively poorly understood. The fact that the Y870A mutant binds to its luminal chaperones, and forms TLR9 dimers suggests that it has retained at least its luminal integrity. Perhaps the properly folded and dimerized luminal domain is unable to be unfolded by the ER quality control machinery—a requirement for retrotranslocation of ERAD substrates into the cytosol for proteosomal degradation—obviating the need for a distinct disposal mechanism, much like an aggregate.

While traditionally associated with ligand-induced signaling, we show here that the TIR domain is important for stability and normal receptor trafficking. This is the first report, to our knowledge, implicating this region in receptor stability in the ER. It will be of interest to investigate how this conserved tyrosine residue impacts the stability and trafficking of other TLRs and adaptor molecules, as many of these molecules have specific subcellular localization requirements. Furthermore, the Y870A mutant TLR9 model may provide an interesting platform to better characterize the autophagosomal degradation pathway of integral membrane protein disposal.

## Materials and methods

### Mice

C57BL/6 mice and *Tlr9*^*-/-*^ mice [33] were housed in our AAALAC certified animal facility. Mice were used in experiments between 7 and 10 weeks of age. Mice were euthanized using CO2 asphyxiation. All experiments were performed with approval of the Children’s Hospital of Philadelphia Institutional Animal Care and Use Committee protocol number 921.

### Antibodies and reagents

The following Western blot antibodies were purchased from Cell Signaling: rabbit anti-HA (clone C29F4), mouse anti-FLAG (clone 9A3), mouse anti-GFP (clone 4B10), rabbit anti-phospo-Erk (clone D13.14.4E), mouse anti-Total Erk (clone L34F12), and mouse anti-IκBα (clone 44D4). Secondary antibodies (mouse and rabbit IgG) were purchased from Licor.

Antibodies used for flow cytometry from BD include TNFα (clone MP6-XT22) conjugated to Alexa Fluor 700, IL-6 (clone MP5-20F3) conjugated to PE, and CD11c (clone HL3) conjugated to PeCy7. HA (clone 6E2) conjugated to Alexa Fluor 647 was purchased from Cell Signaling.

Antibodies used for microscopy include anti-HA antibody (product no. 11 867 423 001, Roche), mouse anti-LAMP1 antibody-lysosomal marker (product no. sc-18821, Santa Cruz Biotechnoogy), mouse anti-GM130 antibody-Cis-golgi marker (product no. 610822, BD Transduction laboratories), Rabbit anti-calreticulin antibody-ER marker (product no. ab 2907, abcam), mouse anti-SQSTM1 / p62 antibody- autophagy marker (product no. ab 56416, abcam) and Rabbit anti-LC3b antibody-autophagy marker (product no. 3868, Cell signaling Technology). The respective secondary antibodies used were goat anti-rat-alexa fluor 568 (product no. A-11077), and Goat anti-Rb-alexa flour 488 (product no. A1126) and Goat anti-mouse-alexa flour 488 (product no. A28175) obtained from Invitrogen/Thermofisher Scientific.

### Plasmids and cloning

pUNO-mTLR9-HA was purchased from InvivoGen. Tyrosine 870 of TLR9 was mutated to either a phenylalanine (Y870F) or alanine (Y870A) using the QuikChange Lightning Site-Directed Mutagenesis Kit (Agilent Technologies) according to the manufacturer’s instructions. The following primers were used. Y870F Forward: 5’-CGCCCAAACTCTCCCTTTTGATGCCTTCGTGG-3’; Y870F Reverse: 5’-CCACGAAGGCATCAAAAGGGAGAGTTTGGGCG-3’; Y870A Forward: 5’-GCAGCGCCCAAACTCTCCCTGCTGA-3’; Y870A Reverse: 5’-CACGAAGGCATCAGCAGGGAGAGTT-3. The primer used for sequencing TLR9 was 5’-TGCTTTGGCCTTTCACTCTT-3’. WT-TLR9-HA, TLR9 Y870F-HA, and TLR9 Y870A-HA were cut from the pUno vector with the restriction enzymes Age1 and Hpa1 (New England Biolabs) and sub-cloned into the MIGR1 retrovirus (a kind gift from W. Pear, University of Pennsylvania) multiple cloning site (MCS) upstream of the internal ribosomal entry site. For this, Age1 was inserted into the MCS of MIGR1 using a BglII-Age1-Hpa1 DNA linker.

Tyrosine870 of GFP-tagged TLR9 in the GFP-N1 expression vector (a kind gift from A. Iwasaki, Yale) was mutated to alanine as stated above (Y870A-GFP). Flag-tagged UNC93B1 (in pCDNA3.1) was a kind gift from G.M. Barton, Berkeley.

A transgene expressing the tetR with a human c-Myc nuclear localization signal (nls) was engineered downstream of and in frame with the eGFP and *Thosea asigna* virus 2A peptide sequence followed by the insertion of an internal ribosome entry sequence (IRES) and various mouse *TLR9* sequences which contained at the 3’-terminus the influenza hemagglutinin epitope tag (HA) [34–38]. Driving the transgenes was the human CMV immediate early gene promoter (CMV) in which two tetO sequences were located downstream of the TATA box with the objective to repress and regulate expression to improve the ability to follow the subcellular location of the HA tagged TLR9. Construction of the transgene used standard molecular biology procedures with complete sequence confirmation.

### Culture and retroviral transduction of mouse BMDCs

Bone marrow was flushed from the tibias and femurs of C57BL/6 and *Tlr9*^*-/-*^ mice and cultured for 10 days in IMDM medium (Invitrogen) containing antibiotics (penicillin, streptomycin, and glutamine), 10% fetal bovine serum, and GM-CSF (3.3ng/ml; Peprotech). MIGR1 retrovirus expressing WT TLR9, TLR9 Y870F, or TLR9 Y870A was constructed using the calcium phosphate method of transfection into HEK293T cells. Briefly, HEK293T cells (5 x 10^6^) were seeded on a 10-cm tissue culture plate. The following day, cells were transfected with MIGR1 plasmid expressing WT TLR9, Y870F, or Y870A along with the ecotropic packaging vectors pCGP and pHIT123 (a kind gift from W. Pear). Twenty-four hours after transfection, supernatant was collected, mixed with polybrene to a final concentration of 4μg/ml, and plated onto cultured bone marrow cells in a 6 or 24-well plate. Plates were centrifuged for 2 hours at 2000rpm at 32 degrees Celsius. After transduction, cells were replaced with fresh DC media, and plates were returned to 37 degrees Celsius. Bone marrow cells were transduced again the following day by the same method. Transduction was assessed by GFP expression (flow cytometry) or by HA expression (flow cytometry and Western blot). After transduction, cells were differentiated to DCs for an additional 7–8 days with GM-CSF. Once differentiated, transduced BMDCs were used in various assays.

### Biochemical assessment of cellular localization of TLR9

Seven to eight days after transduction of WT or Y870A TLR9 into *Tlr9*^*-/-*^ KO BMDCs, the cells were incubated either with an inhibitor of post-golgi transport, Brefeldin A (Sigma) or an inhibitor of endocytosis, Bafilomycin A (Sigma) at a final concentration of 7nM and 0.5μM, respectively, for 4 hours. To determine temperature-sensitive TLR9 trafficking these cells were also grown overnight in incubators set at 30°C prior to their incubation with CpG for 4 hours. The whole cell lysates were assessed by Western blot for TLR9 expression pattern, and the culture media were evaluated for cytokine level.

### Cytokine secretion measurements

After stimulation of cultured BMDCs with CpG (1μg/ml), supernatants were collected and centrifuged for 10 minutes at 13,200rpm at 4°C. Cytokine concentrations were measured in the supernatants using TNFα and IL-6 ELISA kits (BD OptEIA, BD) according to the manufacturer’s instructions. CpG DNA sequence was the standard CpG1826 type B oligomer: 5’-tccatgacgttcctgacgtt-3’. CpG DNA was synthesized by Integrated DNA Technologies (IL, USA), endotoxin free on a phosphorothioate backbone and routinely tested for possible contamination by assay on TLR9-/- cells.

### Western blotting

For preparation of whole cell lysate, cells were washed in cold PBS and lysed in 1% NP40 buffer containing 150mM NaCl, 1M Tris (pH 7.4), complete protease inhibitor cocktail (Sigma), 100mM NaF, 100mM NaVanadate, and 100mM phenylmethanesulfonylflouride. Lysates were centrifuged for 10 minutes at 13,200 rpm and stored at -20°C. All lysates were boiled and separated on SDS-PAGE (10% Bis-Tris gel; Invitrogen) and analyzed by Western blotting. Licor software of analysis was used to analyze blots developed on Odyssey.

### Co-transfection and co-immunoprecipitation

293T cells (2 x 10^6^) were seeded onto 60-mm tissue culture plates. The following day, cells were cotransfected with DNA (4μg each construct) with Lipofectamine (Invitrogen). After 24 hours, lysates were prepared as stated above. For HA pull-downs, lysates were precleared with rabbit IgG beads (Trueblot) and then incubated with HA antibody (1:50, Cell Signaling) for one hour. Following antibody incubation, HA was immunoprecipitated with rabbit IgG beads for 2 hours at 4°C. Beads were washed four times with 1% NP40 buffer. Following the last wash, 2x sample buffer was added to the beads to elute protein, and the beads were boiled for ten minutes. Immunoprecipitated lysates were subjected to Western analysis as described above. For GFP pull-downs, lysates were incubated for 2 hours at 4°C with GFP-Trap (Chromotek), GFP-conjugated agarose beads. Beads were then washed 4 times, and samples were loaded as described above.

### Flow cytometry

For detection of surface CD11c, cells were washed, pelleted and resuspended in FACS buffer (PBS containing 2% fetal bovine serum and 0.01% sodium azide). Cells were then incubated with CD11c antibody conjugated to PeCy7 for 20 minutes. For detection of intracellular cytokines and HA, cells stimulated with CpG in the presence of Brefeldin-A were fixed and permeablized with 1x cytofix/cytoperm (BD) for 20 minutes. Cells were washed with 1x permwash (BD) and stained with TNF antibody conjugated to AF-700, IL-6 antibody conjugated to PE, and HA antibody conjugated to AF-647. All steps were performed at 4°C. Cells were analyzed on a LSR II (BD) using FACSDiva software, and data were analyzed using FlowJo software (TreeStar).

### Immunofluorescence microscopy

TLR9 KO BMDCs were seeded overnight on Poly-L lysine coated coverslips contained in the wells of a 24-well plate (0.5million/well) and were transduced with doxycycline inducible TLR9 retroviruses. Three days post transduction, TLR9 expression was induced using 0.5μg/ml of doxycycline for 4 and 24 hrs. The cells were fixed and permeabilized using the eBioscience Foxp3/transcription factor staining buffer set (product no. 00-5523-00, ThermoFisher Scientific). The cells were then stained with and the respective organelle antibodies. The immunofluorescence images were captured using Leica DM4000B upright scope paired with a Spot RT/SE slider images using 63x objective.

Two complementary methods were used to determine colocalization. First, we computed colocalization using Pearson’s rank correlation of the intensity of all pixels from images of single cells, preceded by thresholding using the automated procedure of Costes performing 100 iterations [24]. This method uses an iterative procedure to determine the threshold for noise determined as the greatest value for which only intensities less than the threshold produces a Pearson’s correlation coefficient of zero. Analysis was performed using the ImageJ (Fiji) colo 2 algorithm. The images were converted to 8 bit greyscale prior to analysis. Second, we assessed the percent area of signal overlap in thresholded images of single cells, independent of variations in signal intensity, using a modification of the method used in Dennis et al., 2015 [39]. Briefly, images were cropped to outline single cells. Binary images from each fluorophore were generated by subtracting the local background using a rolling ball radius of 10 pixels with smoothing disabled, and then manual thresholding. The resulting binary images were multiplied using the Image Calculator function to determine the area of overlap. The areas of overlap and of total labeling for HA in structures larger than 5 pixels were quantified using the Analyze Particles function. Values of percent overlap represent the ratio of the area of overlap to the area of total HA labeling.

### Statistical analysis

Graphs and statistical tests were generated and analyzed using Prism Version 5.0. In general, ANOVA was used to determine significant differences within multiple groups. Bonferroni post-test was then used to test specific pairwise comparisons of interest.

## References

[1] Medvedev AE. Toll-like receptor polymorphisms, inflammatory and infectious diseases, allergies, and cancer. J Interferon Cytokine Res. 2013;33(9):467–84. Epub 2013/05/17. 10.1089/jir.2012.0140 ; PubMed Central PMCID: PMC3760066. 23675778 PMC3760066

[2] Takeda K, Kaisho T, Akira S. Toll-like receptors. Annu Rev Immunol. 2003;21:335–76. Epub 2003/01/14. 10.1146/annurev.immunol.21.120601.141126 [pii]. . 12524386

[3] Brinkmann MM, Spooner E, Hoebe K, Beutler B, Ploegh HL, Kim YM. The interaction between the ER membrane protein UNC93B and TLR3, 7, and 9 is crucial for TLR signaling. J Cell Biol. 2007;177(2):265–75. Epub 2007/04/25. doi: jcb.200612056 [pii] 10.1083/jcb.200612056 ; PubMed Central PMCID: PMC2064135. 17452530 PMC2064135

[4] Kim YM, Brinkmann MM, Paquet ME, Ploegh HL. UNC93B1 delivers nucleotide-sensing toll-like receptors to endolysosomes. Nature. 2008;452(7184):234–8. Epub 2008/02/29. 10.1038/nature06726 [pii]. . 18305481

[5] Ewald SE, Engel A, Lee J, Wang M, Bogyo M, Barton GM. Nucleic acid recognition by Toll-like receptors is coupled to stepwise processing by cathepsins and asparagine endopeptidase. J Exp Med. 2011;208(4):643–51. Epub 2011/03/16. 10.1084/jem.20100682 [pii]. ; PubMed Central PMCID: PMC3135342. 21402738 PMC3135342

[6] Ewald SE, Lee BL, Lau L, Wickliffe KE, Shi GP, Chapman HA, et al The ectodomain of Toll-like receptor 9 is cleaved to generate a functional receptor. Nature. 2008;456(7222):658–62. Epub 2008/09/30. 10.1038/nature07405 [pii]. ; PubMed Central PMCID: PMC2596276. 18820679 PMC2596276

[7] Park B, Brinkmann MM, Spooner E, Lee CC, Kim YM, Ploegh HL. Proteolytic cleavage in an endolysosomal compartment is required for activation of Toll-like receptor 9. Nat Immunol. 2008;9(12):1407–14. Epub 2008/10/22. 10.1038/ni.1669 [pii]. ; PubMed Central PMCID: PMC2735466. 18931679 PMC2735466

[8] Sasai M, Linehan MM, Iwasaki A. Bifurcation of Toll-like receptor 9 signaling by adaptor protein 3. Science. 2010;329(5998):1530–4. Epub 2010/09/18. 10.1126/science.1187029 [pii]. ; PubMed Central PMCID: PMC3063333. 20847273 PMC3063333

[9] Mantegazza AR, Guttentag SH, El-Benna J, Sasai M, Iwasaki A, Shen H, et al Adaptor protein-3 in dendritic cells facilitates phagosomal toll-like receptor signaling and antigen presentation to CD4(+) T cells. Immunity. 2012;36(5):782–94. 10.1016/j.immuni.2012.02.018 ; PubMed Central PMCID: PMCPMC3361531. 22560444 PMC3361531

[10] Ohnishi H, Tochio H, Kato Z, Orii KE, Li A, Kimura T, et al Structural basis for the multiple interactions of the MyD88 TIR domain in TLR4 signaling. Proc Natl Acad Sci U S A. 2009;106(25):10260–5. Epub 2009/06/10. 10.1073/pnas.0812956106 [pii]. ; PubMed Central PMCID: PMC2693180. 19506249 PMC2693180

[11] Li C, Zienkiewicz J, Hawiger J. Interactive sites in the MyD88 Toll/interleukin (IL) 1 receptor domain responsible for coupling to the IL1beta signaling pathway. J Biol Chem. 2005;280(28):26152–9. Epub 2005/04/26. doi: M503262200 [pii] 10.1074/jbc.M503262200 . 15849357

[12] Jiang Z, Georgel P, Li C, Choe J, Crozat K, Rutschmann S, et al Details of Toll-like receptor:adapter interaction revealed by germ-line mutagenesis. Proc Natl Acad Sci U S A. 2006;103(29):10961–6. Epub 2006/07/13. doi: 0603804103 [pii] 10.1073/pnas.0603804103 ; PubMed Central PMCID: PMC1544157. 16832055 PMC1544157

[13] Slack JL, Schooley K, Bonnert TP, Mitcham JL, Qwarnstrom EE, Sims JE, et al Identification of two major sites in the type I interleukin-1 receptor cytoplasmic region responsible for coupling to pro-inflammatory signaling pathways. J Biol Chem. 2000;275(7):4670–8. Epub 2000/02/15. . 10671496 10.1074/jbc.275.7.4670

[14] Medvedev AE, Piao W, Shoenfelt J, Rhee SH, Chen H, Basu S, et al Role of TLR4 tyrosine phosphorylation in signal transduction and endotoxin tolerance. J Biol Chem. 2007;282(22):16042–53. Epub 2007/03/30. doi: M606781200 [pii] 10.1074/jbc.M606781200 ; PubMed Central PMCID: PMC2675888. 17392283 PMC2675888

[15] Lin Z, Lu J, Zhou W, Shen Y. Structural insights into TIR domain specificity of the bridging adaptor Mal in TLR4 signaling. PLoS One. 2012;7(4):e34202 Epub 2012/04/10. 10.1371/journal.pone.0034202 [pii]. ; PubMed Central PMCID: PMC3317499. 22485159 PMC3317499

[16] Latz E, Verma A, Visintin A, Gong M, Sirois CM, Klein DC, et al Ligand-induced conformational changes allosterically activate Toll-like receptor 9. Nat Immunol. 2007;8(7):772–9. Epub 2007/06/19. doi: ni1479 [pii] 10.1038/ni1479 . 17572678

[17] Sinha SS, Cameron J, Brooks JC, Leifer CA. Complex Negative Regulation of TLR9 by Multiple Proteolytic Cleavage Events. J Immunol. 2016;197(4):1343–52. 10.4049/jimmunol.1502357 ; PubMed Central PMCID: PMCPMC4976034. 27421483 PMC4976034

[18] Onji M, Kanno A, Saitoh S, Fukui R, Motoi Y, Shibata T, et al An essential role for the N-terminal fragment of Toll-like receptor 9 in DNA sensing. Nat Commun. 2013;4:1949 10.1038/ncomms2949 . 23752491

[19] Mouchess ML, Arpaia N, Souza G, Barbalat R, Ewald SE, Lau L, et al Transmembrane mutations in Toll-like receptor 9 bypass the requirement for ectodomain proteolysis and induce fatal inflammation. Immunity. 2011;35(5):721–32. 10.1016/j.immuni.2011.10.009 ; PubMed Central PMCID: PMCPMC3230302. 22078797 PMC3230302

[20] Randow F, Seed B. Endoplasmic reticulum chaperone gp96 is required for innate immunity but not cell viability. Nat Cell Biol. 2001;3(10):891–6. Epub 2001/10/05. 10.1038/ncb1001-891 [pii]. . 11584270

[21] Yang Y, Liu B, Dai J, Srivastava PK, Zammit DJ, Lefrancois L, et al Heat shock protein gp96 is a master chaperone for toll-like receptors and is important in the innate function of macrophages. Immunity. 2007;26(2):215–26. Epub 2007/02/06. doi: S1074-7613(07)00116-1 [pii] 10.1016/j.immuni.2006.12.005 ; PubMed Central PMCID: PMC2847270. 17275357 PMC2847270

[22] Liu B, Li Z. Endoplasmic reticulum HSP90b1 (gp96, grp94) optimizes B-cell function via chaperoning integrin and TLR but not immunoglobulin. Blood. 2008;112(4):1223–30. Epub 2008/05/30. 10.1182/blood-2008-03-143107 [pii]. ; PubMed Central PMCID: PMC2515121. 18509083 PMC2515121

[23] Staron M, Yang Y, Liu B, Li J, Shen Y, Zuniga-Pflucker JC, et al gp96, an endoplasmic reticulum master chaperone for integrins and Toll-like receptors, selectively regulates early T and B lymphopoiesis. Blood. 2010;115(12):2380–90. Epub 2009/12/08. 10.1182/blood-2009-07-233031 [pii]. ; PubMed Central PMCID: PMC2845896. 19965672 PMC2845896

[24] Costes SV, Daelemans D, Cho EH, Dobbin Z, Pavlakis G, Lockett S. Automatic and quantitative measurement of protein-protein colocalization in live cells. Biophys J. 2004;86(6):3993–4003. 10.1529/biophysj.103.038422 ; PubMed Central PMCID: PMCPMC1304300. 15189895 PMC1304300

[25] Hasan M, Gruber E, Cameron J, Leifer CA. TLR9 stability and signaling are regulated by phosphorylation and cell stress. J Leukoc Biol. 2016;100(3):525–33. 10.1189/jlb.2A0815-337R . 26957214 PMC6608028

[26] Chaturvedi A, Dorward D, Pierce SK. The B cell receptor governs the subcellular location of Toll-like receptor 9 leading to hyperresponses to DNA-containing antigens. Immunity. 2008;28(6):799–809. 10.1016/j.immuni.2008.03.019 ; PubMed Central PMCID: PMCPMC2601674. 18513998 PMC2601674

[27] Young JC. The role of the cytosolic HSP70 chaperone system in diseases caused by misfolding and aberrant trafficking of ion channels. Dis Model Mech. 2014;7(3):319–29. 10.1242/dmm.014001 ; PubMed Central PMCID: PMCPMC3944492. 24609033 PMC3944492

[28] Kawai T, Akira S. The role of pattern-recognition receptors in innate immunity: update on Toll-like receptors. Nat Immunol. 2010;11(5):373–84. Epub 2010/04/21. doi: ni.1863 [pii] 10.1038/ni.1863 . 20404851

[29] Leifer CA, Brooks JC, Hoelzer K, Lopez J, Kennedy MN, Mazzoni A, et al Cytoplasmic targeting motifs control localization of toll-like receptor 9. J Biol Chem. 2006;281(46):35585–92. Epub 2006/09/23. doi: M607511200 [pii] 10.1074/jbc.M607511200 ; PubMed Central PMCID: PMC2758030. 16990271 PMC2758030

[30] Farinha CM, Canato S. From the endoplasmic reticulum to the plasma membrane: mechanisms of CFTR folding and trafficking. Cell Mol Life Sci. 2017;74(1):39–55. 10.1007/s00018-016-2387-7 . 27699454 PMC11107782

[31] Houck SA, Cyr DM. Mechanisms for quality control of misfolded transmembrane proteins. Biochim Biophys Acta. 2012;1818(4):1108–14. 10.1016/j.bbamem.2011.11.007 ; PubMed Central PMCID: PMCPMC3288195. 22100602 PMC3288195

[32] Pankiv S, Clausen TH, Lamark T, Brech A, Bruun JA, Outzen H, et al p62/SQSTM1 binds directly to Atg8/LC3 to facilitate degradation of ubiquitinated protein aggregates by autophagy. J Biol Chem. 2007;282(33):24131–45. 10.1074/jbc.M702824200 . 17580304

[33] Hemmi H, Takeuchi O, Kawai T, Kaisho T, Sato S, Sanjo H, et al A Toll-like receptor recognizes bacterial DNA. Nature. 2000;408(6813):740–5. Epub 2000/12/29. 10.1038/35047123 . 11130078

[34] Postle K, Nguyen TT, Bertrand KP. Nucleotide sequence of the repressor gene of the TN10 tetracycline resistance determinant. Nucleic Acids Res. 1984;12(12):4849–63. ; PubMed Central PMCID: PMCPMC318884. 6330687 10.1093/nar/12.12.4849 PMC318884

[35] Hillen W, Berens C. Mechanisms underlying expression of Tn10 encoded tetracycline resistance. Annu Rev Microbiol. 1994;48:345–69. 10.1146/annurev.mi.48.100194.002021 . 7826010

[36] Orth P, Schnappinger D, Hillen W, Saenger W, Hinrichs W. Structural basis of gene regulation by the tetracycline inducible Tet repressor-operator system. Nat Struct Biol. 2000;7(3):215–9. 10.1038/73324 . 10700280

[37] Berens C, Hillen W. Gene regulation by tetracyclines. Constraints of resistance regulation in bacteria shape TetR for application in eukaryotes. Eur J Biochem. 2003;270(15):3109–21. . 12869186 10.1046/j.1432-1033.2003.03694.x

[38] Bertram R, Hillen W. The application of Tet repressor in prokaryotic gene regulation and expression. Microb Biotechnol. 2008;1(1):2–16. 10.1111/j.1751-7915.2007.00001.x ; PubMed Central PMCID: PMCPMC3864427. 21261817 PMC3864427

[39] Dennis MK, Mantegazza AR, Snir OL, Tenza D, Acosta-Ruiz A, Delevoye C, et al BLOC-2 targets recycling endosomal tubules to melanosomes for cargo delivery. J Cell Biol. 2015;209(4):563–77. 10.1083/jcb.201410026 ; PubMed Central PMCID: PMCPMC4442807. 26008744 PMC4442807

